# Imaging intralesional heterogeneity of sodium concentration in multiple sclerosis: Initial evidence from ^23^Na-MRI

**DOI:** 10.1016/j.jns.2018.01.027

**Published:** 2018-04-15

**Authors:** James T. Grist, Frank Riemer, Mary A. McLean, Tomasz Matys, Fulvio Zaccagna, Sarah F. Hilborne, Jackie P. Mason, Ilse Patterson, Rhys Slough, Joshua Kaggie, Surrin S. Deen, Martin J. Graves, Joanne L. Jones, Alasdair J. Coles, Ferdia A. Gallagher

**Affiliations:** a Department of Radiology, University of Cambridge, Cambridge, UK; b Cancer Research UK Cambridge Institute, University of Cambridge, Li Ka Shing Centre, Cambridge, UK; c Department of Radiology, Addenbrooke's Hospital, Cambridge, UK; d Department of Clinical Neurosciences, University of Cambridge, Cambridge, UK

**Keywords:** Multiple sclerosis, ^23^Na, Intracellular sodium, Extracellular sodium, High-resolution, 3-tesla

## Abstract

Sodium MRI (^23^Na-MRI) has been used to non-invasively quantify tissue sodium but has been limited by low spatial resolution. Here we demonstrate for the first time that high resolution ^23^Na-MRI reveals the spatial heterogeneity of sodium concentration within a multiple sclerosis (MS) lesion. A patient with treatment-naïve relapsing-remitting MS and a ring-enhancing lesion was imaged using ^23^Na-MRI. The periphery of the lesion demonstrated an elevated total sodium content compared to the normal appearing white and grey matter (p < 0.01), as well as a heterogeneous distribution of both the total tissue sodium concentration and the intracellular-weighted sodium concentration.

## Highlights

• High spatial resolution ^23^Na MRI acquired in a clinically feasible time at 3 T.
• Quantification of the intracellular sodium concentration in an active MS lesion for the first time
• Characterization of a sodium gradient in an active MS lesion

## Graphical abstract

Images and analysis showing the heterogeneous distribution of sodium in an active Multiple Sclerosis lesion.

A & B show total and intracellular weighted sodium concentration maps of the MS brain, respectively. C shows regional differences in sodium distribution relative to normal appearing brain and D reveals a spatially dependent distribution of sodium in an active MS lesion.

**Figure:**
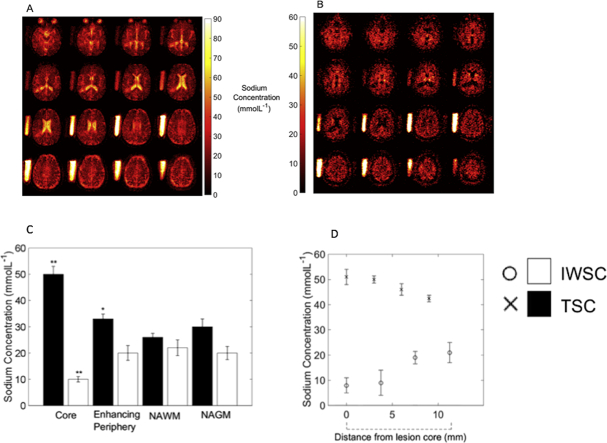

## 1 Introduction

Sodium MRI is a powerful tool for quantitatively assessing the distribution of tissue sodium [1], [2], [3]. The total tissue sodium concentration, a combined measure of the intracellular and extracellular sodium, is increased in MS and correlates with the severity of disability [4], [5], [6]. This increase in sodium may reflect the underlying degree of inflammation and mitochondrial dysfunction. Until recently, sodium MRI has been limited by low resolution, however recent advances in pulse sequence design and high-performance gradient systems have enabled increased resolution at clinical field strengths, allowing the spatial heterogeneity of sodium distribution to be studied non-invasively. Using this approach, we have imaged the distribution of sodium in a patient with MS to study the spatial variation of tissue sodium across a gadolinium-enhancing lesion. The results show, for the first time, a gradient of the total and intracellular-weighted sodium across the lesion, and demonstrate the potential of the technique to probe tissue heterogeneity in MS.

## 2 Methods

### 2.1 Patient and imaging

A 27 year old female was diagnosed with MS in November 2014 having presented with two brainstem episodes consisting of diplopia and ataxia, as well as right hemisensory symptoms during the second episode. In April 2016, she developed a third episode consisting of numbness affecting the right thigh, back, and right side of her face. When she was reviewed a month later, her symptoms had resolved and she declined disease-modifying therapy. The participant was enrolled into a study approved by a local ethical review committee following her written informed consent, NRES Committee East of England, Cambridge South, REC number 15/EE/0255. An MRI in September 2016 showed gadolinium-enhancing lesions including a 24 mm ring-enhancing mass in the right temporo-occipital lobe; ^23^Na-MRI was performed subsequently. All imaging in this study was performed in accordance with local rules and regulations.

Imaging was performed on a 3 T MRI system (MR750, GE Healthcare, Waukesha, WI) using a ^1^H/^23^Na dual-tuned head coil (Rapid Biomedical, Rimpar). Proton (^1^H) T_1_-weighted images (T_1_W) were acquired using an inversion prepared 3D GRE sequence with: inversion time (TI) 450 ms; field of view (FOV) 240 mm; flip angle 12°; repetition time (TR) 8.14 ms; echo time (TE) 3.192 ms, resolution 1 × 1 × 2 mm.

Total sodium imaging was acquired using a 3D cones sequence [7], [8]: FOV 240 mm; TE 0.5 ms; TR 100 ms; flip angle 90°; averages 3; resolution 3 mm isotropic; scan time 12 min. Intracellular-weighted sodium imaging was acquired as above, utilizing an inversion pulse: inversion time (TI) 30 ms, resolution 3.75 mm isotropic [9]. Sodium calibration phantoms were used for quantification of the images (20, 100 mM NaCl in 4% agar). T_1_W imaging was then acquired again following 0.1 mL/kg gadobutrol (Bayer, Germany).

### 2.2 Quantitative analysis

**Fig. 1:**
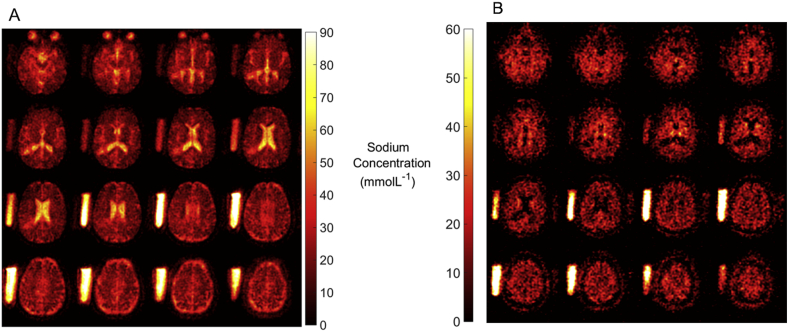
Example images of total sodium and intracellular weighted sodium in the MS brain. A) Total sodium concentration map; B) Intracellular weighted sodium concentration map.

Intracellular-weighted sodium concentration (IWSC) and total sodium concentration (TSC) maps were noise-corrected using a power correction method by defining the noise baseline from the background. Voxels were fitted against a linear calibration curve derived from the sodium phantoms and selected TSC and IWSC images are shown in Fig. 1A and B, respectively [10], [11].

Volumetric analysis was performed using Osirix Lite (v7.5, Pixmeo). The periphery of the lesion was defined as the enhancing rim following gadolinium administration. Sodium maps were co-registered to the contrast-enhanced T_1_-weighted images using SPM 12 (UCL). Regions of interest (ROIs) were drawn on contrast-enhanced T_1_W images in the lesion core, the enhancing periphery, and the normal appearing white and grey matter (NAWM and NAGM). To assess the presence of a sodium gradient, concentric radial shells, formed from 8 equally spaced spokes projected from the lesion centre to periphery, were formed and the average sodium concentration in each volume calculated. Analysis was performed over eight slices.

A two-sided ANOVA was performed for both IWSC and TSC groups using a Tukey *post hoc* analysis. Two-tailed *t*-tests were then used to assess significance p < 0.05 (Matlab, Mathworks, MA).

## 3 Results

**Fig. 2:**
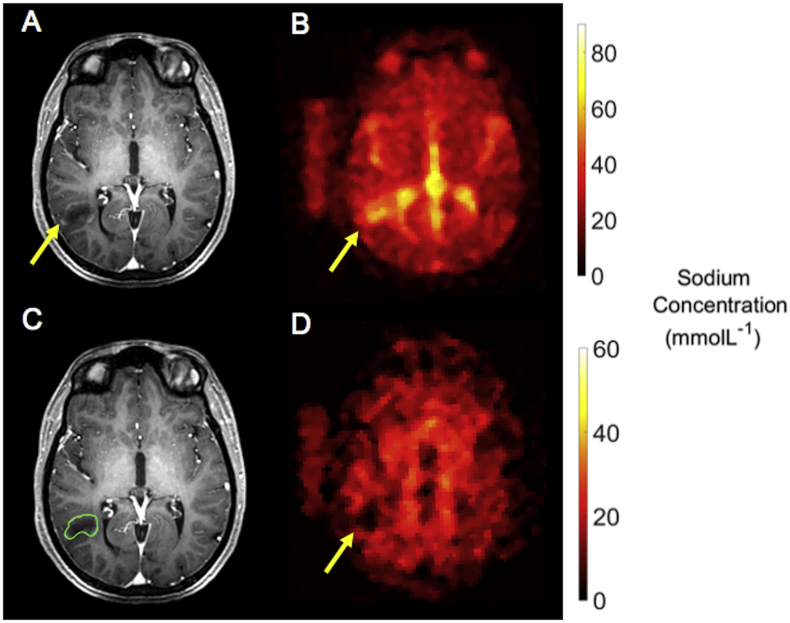
^1^H and ^23^Na images of the MS lesion and quantitative sodium analysis of the MS brain. A) Post contrast-enhanced T_1_-weighted image; B) Total sodium map; C) ROI placement on enhancing lesion; D) Intracellular weighted (fluid suppressed) sodium map. Arrow denotes active MS lesion.

The ring-enhancing lesion, identified on ^1^H post contrast T_1_W imaging, measured 6.9 cm^3^ and was selected for heterogeneity analysis. Selected images are shown in Fig. 2A–D.

**Fig. 3:**
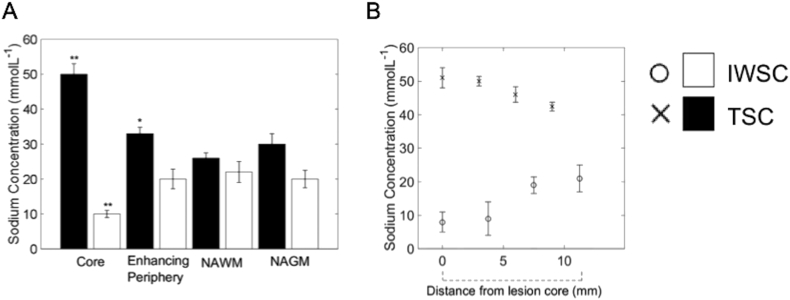
Quantitative analysis of heterogeneous sodium distribution in a large MS lesion. A) Differences between core, enhancing periphery, NAWM, and NAGM (measurements over 8 slices). *Significant in comparison to NAWM, p < 0.01, and NAGM, p < 0.05. **Significant in comparison to NAWM and NAGM, p < 0.01. B) Average IWSC and TSC gradient in MS lesion.

The TSC in the core of the lesion was significantly higher, and had a significantly lower IWSC (TSC: 50 ± 1 mmol L^− 1^, IWSC: 10 ± 1 mmol L^− 1^) compared to both NAWM and NAGM (TSC: 26 ± 1, 30 ± 2 mmol L^− 1^ and IWSC: 22 ± 3, 20 ± 2 mmol L^− 1^ respectively; p < 0.01 for all comparisons; Fig. 3A). However, the periphery of the lesion showed a higher total sodium concentration, but no discernible difference in IWSC (TSC: 33 ± 2 mmol L^− 1^, IWSC: 20 ± 4 mmol L^− 1^) in comparison to both NAWM and NAGM (p < 0.05 and p > 0.05 respectively). A marked gradient in the TSC was observed from the lesion core to the periphery ranging from 50 ± 1 mmol L^− 1^ centrally and decreasing to 33 ± 2 mmol L^− 1^ over 12 mm from the centre of the lesion as seen in Fig. 3B; the opposite change in IWSC also seen from the centre to the periphery of the lesion ranging from 8 ± 3 mmol L^− 1^ centrally and increasing to 21 ± 4 mmol L^− 1^.

## 4 Discussion

This work demonstrates the feasibility of utilizing quantitative sodium imaging to study tissue heterogeneity in MS. Recent hardware and software developments have made this study possible, enabling high resolution ^23^Na-MRI acquisition in a clinically acceptable time frame and at routine clinical field strength, as shown in Fig. 1, Fig. 2.

The results demonstrate intralesional sodium heterogeneity with both TSC and IWSC imaging for the first time: the centre of the active plaque had a lower IWSC and an associated higher TSC compared to the enhancing periphery. In contrast, the periphery of the active lesion demonstrated a higher TSC, but no discernible change in the IWSC in comparison to NAWM and NAGM. These regional differences in TSC may represent inflammatory demyelination, oedema or a redistribution of axonal sodium channels. The low IWSC in the lesion centre may represent an increase in cellular necrosis compared to the viable periphery.

It has been noted in previous sodium MRI studies of MS patients that there is a global increase in sodium concentration within both NAWM and NAGM [6]. This may partly relate to changes in sodium channel concentration and their distribution in both astrocytes and neurons [12]. These effects on the transporters may be compounded by functional alterations in the opening and closing thresholds for these channels; for example, systemic thermoregulatory changes seen in MS may affect channel activity [13]. Secondary effects of an abnormal escalation of intra-axonal sodium concentration include activation and reversal of the sodium-calcium exchanger, with increased intra-axonal calcium concentration leading to glutamate release [14]. This in turn can induce oxidative stress and additional structural damage. Furthermore, it has been postulated that a high salt diet may increase the rate of cell death and exacerbation of MS symptoms, which if true, would provide additional support for the role of sodium in the pathogenesis of the disease; however, there is conflicting evidence for this hypothesis in the literature [14], [15].

Given the high spatial resolution of sodium distribution achieved in this study and the demonstration of regional heterogeneity, it may be possible in the future to correlate the neuroanatomical location of altered sodium with an individual patient's symptoms or to predict future symptoms in the absence of an anatomical abnormality on MRI. Therefore, these results demonstrate the possibility of using sodium MRI to probe the biology of MS in new ways in the future.

Although sodium MRI is currently a research tool, translation to routine clinical use could be easily achieved. For example, the dedicated hardware is commercially available and acquisition protocols for imaging sodium are publicly available. Importantly, relatively recent hardware and software developments have made this study possible and we have demonstrated the acquisition of high resolution sodium data in a clinically feasible time frame using a clinical field strength magnet (3 T). With the increased interest in multinuclear MRI for neuroimaging, sodium MRI could become a more routine tool if clinical benefit can be demonstrated.

## Competing financial interests

We declare no competing financial interests.

## Author contributions

Data collection: JG.

Data analysis: JG.

Manuscript preparation: JG, FR, FAG.

Reviewing of manuscript: JG, FR, MAM, TM, FZ, SH, JM, IP, RS, JK, SSD, MJG, JLJ, AC, FAG.
